# Targeting matrix metalloproteinases with novel diazepine substituted cinnamic acid derivatives: design, synthesis, in vitro and in silico studies

**DOI:** 10.1186/s13065-018-0411-8

**Published:** 2018-04-20

**Authors:** Dharmender Rathee, Viney Lather, Ajmer Singh Grewal, Harish Dureja

**Affiliations:** 1 Department of Pharmaceutical Sciences, Maharshi Dayanand University, Rohtak, Haryana 124001 India; 2 Department of Pharmaceutical Chemistry, JCDM College of Pharmacy, Sirsa, Haryana 125055 India; 3 Chitkara College of Pharmacy, Chitkara University, Rajpura, Patiala, Punjab 140401 India

**Keywords:** Targeting, MMP-2, MMP-9, Diazepine, Cinnamic acid, Molecular docking

## Abstract

Lung cancer is the notable cause of cancer associated deaths worldwide. Recent studies revealed that the expression of matrix metalloproteinases (MMPs) is extremely high in lung tumors compared with non-malignant lung tissue. MMPs (-2 and -9) play an important part in tumor development and angiogenesis, which suggests that creating potent MMP-2 and -9 inhibitors, should be an important goal in lung cancer therapy. In the present study, an effort has been made to develop new anti-metastatic and anti-invasive agents, wherein a series of novel diazepine substituted cinnamic acid derivatives were designed, synthesized and assayed for their inhibitory activities on MMP-2 and MMP-9. These derivatives were prepared via microwave assisted reaction of tert-butyl (3-cinnamamidopropyl)carbamate derivatives mixed with 2,3-dibromopropanoic acid and potassium carbonate was added to obtain 4-(tert-butoxycarbonyl)-1-cinnamoyl-1,4-diazepane-2-carboxylic acid derivatives. The newly synthesized compounds were characterized by IR, NMR and mass spectroscopy. All the tested compounds showed good to excellent cytotoxic potential against A549 human lung cancer cells. The active compounds displaying good activity were further examined for the inhibitory activity against MMPs (-2 and -9). In addition, the structure and anticancer activity relationship were further supported by in silico docking studies of the active compounds against MMP-2 and MMP-9.
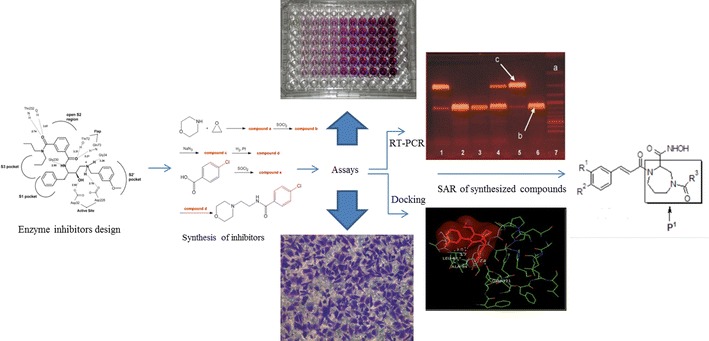

## Introduction

Malignant properties of lung polyp cells, such as metastasis, tissue invasion, irregular tumor growth, tissue remodeling and inflammation, are linked with reformed proteolysis [3, 22]. Matrix metalloproteinases (MMPs) exemplify the most significant group of proteinases, which gets activated directly by degrading the extracellular matrix (ECM) and/or other secreted proteins of the lungs. Conversely, by altering the properties of the cleaved proteins in the alveolar space, MMPs function independently of their proteolytic activity [27]. MMPs are zinc-dependent endopeptidases [5] commonly known as matrixins, which play a special role during tissue remodeling and organ development [18, 34]. Aberration in the expression of MMP is associated with a variety of diseases from respiratory to autoimmune disorder and even cancer, particularly lung cancer. MMPs are known to influence lung cancer metastatic properties and involved several signalling pathways [16]. MMP-2 and -9; gelatinases, are very closely associated with the metastatic properties of lung cancer [39], which suggests that creating potent MMP-2 and MMP-9 inhibitors should be an important goal in lung cancer therapy [31].

**Fig. 1:**
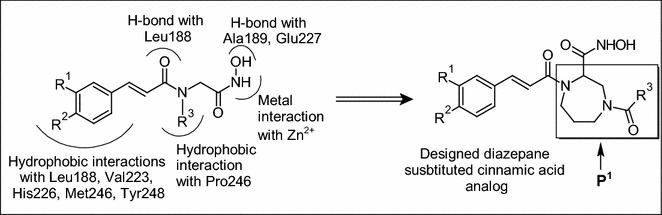
General structure of the designed diazepine substituted cinnamic acid molecule

In the current study, we have used fragment linking and structure based approaches for the design of diazepine substituted cinnamic acid molecule as it involves two (or more) fragments, and extended P1′ group. The fragments which are active against one receptor are joined together to give a higher affinity molecule and the cinnamic acid amides with extended P1′ group could further increase the activity. In SAR studies a standard nomenclature Pn, … P1, P2, P3 etc. is used to designate amino acid residues of a peptide substrate (Example of P1 group such as branched alkanes and cycloalkanes) [1, 10]. Various reports have shown diazepine and caffeic acid (hydroxycinnamic acid) derivatives as the active moieties against MMPs [14, 24, 28, 29, 33, 36]. Several modified caffeic acid amides have more steady features [25]. These results encouraged us to design and synthesize a novel series of diazepine substituted cinnamic acid derivatives to explore their inhibitory activity on MMP-2 and MMP-9 (Fig. 1) and their structure–activity relationship (SAR) analysis.

## Materials and methods

### Chemicals

All the chemicals were purchased from Thermo Fisher Scientific and were used as such for the experiments. Melting points were determined using Veego VMP-D melting point apparatus. Thin layer chromatography (Merck silica gel—G) was used to monitor the reaction progress. ^1^H and ^13^C NMR spectra were recorded by Bruker Avance II 300 MHz NMR spectrometer using DMSO-d6 as solvent and are expressed in parts per million (δ, ppm) downfield from tetramethylsilane (internal standard). NMR data is given as multiplicity (s, singlet; d, doublet; t, triplet; m, multiplet) and number of protons. Infrared (IR) spectra were recorded by KBr disc method on a Shimadzu IR affinity FTIR spectrophotometer. The wave number is given in cm^−1^. Mass spectra were taken on Waters, Q-TOF Micromass spectrometer (ESI–MS).

### Synthesis of diazepine substituted cinnamic acid derivatives

Benzaldehyde derivative (1.0 molar eq.) and malonic acid (2.2 molar eq.) were added to 50 mL of dry pyridine, containing (0.015 molar eq.) of aniline, to form a solution. This solution was allowed to stand overnight, followed by heating for 3 h at 55 °C in order to remove carbon dioxide. Reaction mixture was then poured into the mixture of 60 mL of concentrated hydrochloric acid and 100 g of chopped ice. The acid precipitated immediately and then allowed to stand for few minutes for complete separation. The filtration was done followed by washing of product with 10 mL of 5% hydrochloric acid and then with two portions of 10 mL water. At the end, drying of residue was carried out. Cinnamic acid derivatives obtained above (1.0 molar eq.) were refluxed with thionyl chloride (1.1 molar eq.) for 4 h in order to obtain the corresponding acid chlorides. Henceforth, the acid chlorides obtained above were refluxed with tert-butyl (3-aminopropyl)carbamate (1:1) for 4 h and respective tert-butyl (3-cinnamamidopropyl)carbamate derivatives were synthesized. Respective tert-butyl (3-cinnamamidopropyl)carbamate derivatives (1.0 mmol) were mixed with 2,3-dibromopropanoic acid (1.1 mmol) and potassium carbonate (1.1 mmol) was added to obtain 4-(tert-butoxycarbonyl)-1-cinnamoyl-1,4-diazepane-2-carboxylic acid derivatives. This step was performed under microwave irradiation at temperature of 120 °C and power at 90 W for 20 min. The extraction of organic portion was carried out with ethyl acetate. The solvent was removed and the product was recrystallized [11]. Then, to a solution, of synthesized 4-(tert-butoxycarbonyl)-1-cinnamoyl-1,4-diazepane-2-carboxylic acid derivatives (1.0 molar eq.) and methanol (36.72 molar eq.), thionyl chloride (5.0 molar eq.) was added dropwise (at room temperature). After that, stirring was performed overnight to synthesize 1-tert-butyl 3-methyl 4-cinnamoyl-1,4-diazepane-1,3-dicarboxylate derivatives and dried by the agency of rotary evaporator. Various acyl and aryl acid chlorides were refluxed with 1-tert-butyl 3-methyl 4-cinnamoyl-1,4-diazepane-1,3-dicarboxylate derivatives (1:1) for 4 h to get 4-acyl substituted methyl-1-cinnamoyl-1,4-diazepane-2-carboxylate derivatives. In the last step, 4-acyl substituted methyl-1-cinnamoyl-1,4-diazepane-2-carboxylate derivatives were stirred with hydroxylamine (1:1), in methanol for 15 min to obtain the corresponding final products [4, 7, 8, 11, 19]. The reaction products were poured into crushed ice and precipitates which separated out were filtered, dried and recrystallized from ethanol. The synthesis was monitored by TLC on silica gel G Plates.

#### 4-Benzoyl-1-cinnamoyl-*N*-hydroxy-1,4-diazepane-2-carboxamide (1)

Mp (°C) 210; Yield—68.2%; IR (KBr pellets, cm^−1^): 1174 (C–O), 1288 (–C–N), 1423 (–C–H), 1529 (C=C), 1614 (C=N), 1791 (C=O), 2094 (C≡C), 2324 (C≡N), 2677 (–C–H=O), 2742 (–C–H=O), 2937 (C–H), 2976 (=C–H), 3064 (=C–H), 3431 (N–H), 3712 (O–H); ^1^H NMR (DMSO-*d*_6_, δ ppm): 2.010 (s, 1H, OH of NHOH), 8.112 (s, 1H, NH of CONHOH), 3.213–5.161 (m, 9H, diazepane), 7.334–7.643 (m, 10H, CH of C_6_H_5_), 7.337 (s, 1H, CH of ethylene), 7.176 (s, 1H, CH of ethylene); ^13^CNMR (DMSO-*d*_6_, δ ppm): 166.29 (C=O of COC_6_H_5_), 167.75 (C=O of CONHOH), 167.49 (C=O of amide), 144.54 (CH of ethylene), 136.15 (C of phenyl), 129.02 (CH of phenyl), 126.96 (CH of phenyl), 127.88 (CH of phenyl), 130.23 (CH of phenyl), 130.00 (CH of phenyl), 134.57 (C of COC_6_H_5_), 125.92 (CH of COC_6_H_5_), 130.50 (CH of COC_6_H_5_), 129.72 (CH of COC_6_H_5_), 128.66 (CH of COC_6_H_5_), 125.96 (CH of COC_6_H_5_), 128.66 (CH of ethylene), 52.98 (CH, diazepane), 44.87 (CH_2_, diazepane), 40.64 (CH_2_, diazepane), 28.52 (CH_2_, diazepane), 40.01 (CH_2_, diazepane).

#### 4-Butyryl-1-cinnamoyl-*N*-hydroxy-1,4-diazepane-2-carboxamide (2)

Mp (°C) 121–123; Yield—68.9%; IR (KBr pellets, cm^−1^): 1172 (C–O), 1249 (–C–N), 1431 (–C–H), 1581 (C=C), 1625 (C=N), 1707 (C=O), 1724 (C=O), 2133 (C≡C), 2241 (C≡N), 2692 (–C–H=O), 2877 (C–H), 3049 (=C–H), 3068 (=C–H), 3201 (≡C–H), 3408 (N–H), 3712 (O–H); ^1^H NMR (DMSO-*d*_6_, δ ppm): 1.961 (s, 1H, OH of NHOH), 8.104 (s, 1H, NH of CONHOH), 1.685–5.261 (m, 9H, diazepane), 6.945–7.961 (m, 5H, CH of C_6_H_5_), 7.486 (s, 1H, CH of ethylene), 6.985 (s, 1H, CH of ethylene), 0.965 (m, 3H, CH_3_ of COC_3_H_7_), 1.684 (m, 2H, CH_2_ of COC_3_H_7_), 2.780 (m, 2H, CH_2_ of COC_3_H_7_); ^13^CNMR (DMSO-*d*_6_, δ ppm): 167.70 (C=O of COC_3_H_7_), 167.46 (C=O of CONHOH), 166.27 (C=O of amide), 144.54 (CH of ethylene), 136.15 (C of phenyl), 132.37 (CH of phenyl), 131.23 (CH of phenyl), 131.15 (CH of phenyl), 130.50 (CH of phenyl), 127.46 (CH of phenyl), 128.66 (CH of ethylene), 61.45 (CH, diazepane), 52.98 (CH_2_, diazepane), 44.87 (CH_2_, diazepane), 28.90 (CH_2_, diazepane), 40.61 (CH_2_, diazepane), 39.39 (CH_2_ of COC_3_H_7_), 28.52 (CH_2_ of COC_3_H_7_), 17.66 (CH_3_ of COC_3_H_7_). MS ES + (ToF): m/z 360.4.

#### 4-Acetyl-1-cinnamoyl-*N*-hydroxy-1,4-diazepane-2-carboxamide (3)

Mp (°C) 164; Yield—67.8%; IR (KBr pellets, cm^−1^): 1174 (C–O), 1288 (–C–N), 1423 (–C–H), 1529 (C=C), 1614 (C=N), 1791 (C=O), 2094 (C≡C), 2324 (C≡N), 2677 (–C–H=O), 2742 (–C–H=O), 2937 (C–H), 2976 (=C–H), 3064 (=C–H), 3431 (N–H), 3712 (O–H); ^1^H NMR (DMSO-*d*_6_, δ ppm): 2.012 (s, 1H, OH of NHOH), 8.114 (s, 1H, NH of CONHOH), 1.712–5.161 (m, 9H, diazepane), 6.562–7.534 (m, 5H, CH of C_6_H_5_), 7.486 (s, 1H, CH of ethylene), 6.985 (s, 1H, CH of ethylene), 2.732 (m, 3H, CH_3_ of COCH_3_); ^13^CNMR (DMSO-*d*_6_, δ ppm): 172.52 (C=O of COCH_3_), 171.12 (C=O of CONHOH), 164.42 (C=O of amide), 144.48 (CH of ethylene), 138.59 (C of phenyl), 133.77 (CH of phenyl), 135.82 (CH of phenyl), 134.82 (CH of phenyl), 133.27 (CH of phenyl), 133.06 (CH of phenyl), 130.84 (CH of ethylene), 35.46 (CH_2_, C_6_ of diazepane), 45.98 (CH_2_, C_7_ of diazepane), 26.95 (CH_3_ of COCH_3_); MS ES + (ToF): m/z 332.3.

#### 1-Cinnamoyl-*N*-hydroxy-4-propionyl-1,4-diazepane-2-carboxamide (4)

Mp (°C) 190; Yield—69.2%; IR (KBr pellets, cm^−1^): 1172 (C–O), 1286 (–C–N), 1394 (–C–H), 1435 (–C–H), 1546 (C=C), 1629 (C=N), 1707 (C=O), 1714 (C=O), 1737 (C=O), 2135 (C≡C), 2239 (C≡N), 2681 (–C–H=O), 2744 (–C–H=O), 2935 (C–H), 3421 (N–H), 3433 (N–H), 3687 (O–H), 3711 (O–H); ^1^H NMR (DMSO-*d*_6_, δ ppm): 1.985 (s, 1H, OH of NHOH), 8.015 (s, 1H, NH of CONHOH), 2.712–5.161 (m, 9H, diazepane), 7.355–7.523 (m, 5H, CH of C_6_H_5_), 7.334 (s, 1H, CH of ethylene), 6.981 (s, 1H, CH of ethylene), 1.112 (m, 3H, CH_3_ of COC_2_H_5_), 2.112 (m, 2H, CH_2_ of COC_2_H_5_); ^13^CNMR (DMSO-*d*_6_, δ ppm): 158.82 (C=O of COC_2_H_5_), 167.72 (C=O of CONHOH), 168.25 (C=O of amide), 138.08 (CH of ethylene), 134.81 (C of phenyl), 130.82 (CH of phenyl), 124.53 (CH of phenyl), 122.53 (CH of phenyl), 132.84 (CH of phenyl), 130.79 (CH of phenyl), 128.66 (CH of ethylene), 40.05 (CH, diazepane), 32.70 (CH_2_, diazepane), 32.63 (CH_2_, diazepane), 24.04 (CH_2_, diazepane), 40.01 (CH_2_, diazepane), 10.01 (CH_2_ of COC_2_H_5_), 24.11 (CH_3_ of COC_2_H_5_).

#### 4-Acetyl-*N*-hydroxy-1-(3-(3-hydroxyphenyl)acryloyl)-1,4-diazepane-2-carboxamide (5)

Mp (°C) 123; Yield—67.1%; IR (KBr pellets, cm^−1^): 1217 (C–O), 1394 (–C–H), 1436 (–CH3), 1581 (–C–H), 1622 (C=N), 1747 (C=O), 1793 (C=O), 1865 (C–H), 2135 (C≡C), 2239 (–C≡N), 2738 (–CHO), 2758 (–CHO), 2945 (C–H), 2966 (=C–H), 3190 (≡C–H), 3446 (N–H), 3709 (O–H); ^1^H NMR (DMSO-*d*_6_, δ ppm): 2.010 (s, 1H, OH of NHOH), 8.112 (s, 1H, NH of CONHOH), 2.780–5.161 (m, 9H, diazepane), 7.334–7.535 (m, 4H, CH, aromatic), 7.334 (s, 1H, CH of ethylene), 7.176 (s, 1H, CH of ethylene), 1.892 (m, 3H, CH_3_ of COCH_3_), 5.321 (s, H, aromatic OH); ^13^CNMR (DMSO-*d*_6_, δ ppm): 158.58 (C=O of COCH_3_), 167.72 (C=O of CONHOH), 168.25 (C=O of amide), 138.08 (CH of ethylene), 134.81 (C of phenyl), 132.34 (CH of phenyl), 130.82 (C of phenyl), 124.15 (CH of phenyl), 122.34 (CH of phenyl), 122.53 (CH of phenyl), 130.29 (CH of ethylene), 40.06 (CH, diazepane), 39.68 (CH_2_, diazepane), 39.22 (CH_2_, diazepane), 24.04 (CH_2_, diazepane), 40.02 (CH_2_, diazepane), 24.11 (CH_3_ of COCH_3_).

#### *N*-Hydroxy-1-(3-(3-hydroxyphenyl)acryloyl)-4-propionyl-1,4-diazepane-2-carboxamide (6)

Mp (°C) 125–127; Yield—69.4%; IR (KBr pellets, cm^−1^):1170 (C–O), 1396 (C–H), 1438 (CH_3_), 1627 (C=N), 2133 (C≡C), 2239 (C≡N), 2947 (C–H), 3057 (=C–H), 3136 (≡C–H), 3452 (N–H), 3765 (O–H); ^1^H NMR (DMSO-*d*_6_, δ ppm): 1.984 (s, 1H, OH of NHOH), 8.015 (s, 1H, NH of CONHOH), 5.386 (H, OH, aromatic), 3.134–5.016 (m, 9H, diazepane), 6.945–7.535 (m, 4H, CH of C_6_H_5_), 7.334 (s, 1H, CH of ethylene), 7.176 (s, 1H, CH of ethylene), 1.235 (m, 2H, CH_2_ of COCH_2_CH_3_), 2.235 (m, 3H, CH_3_ of COCH_2_CH_3_); ^13^CNMR (DMSO-*d*_6_, δ ppm): 168.25 (C=O of COCH_2_CH_3_), 167.72 (C=O of CONHOH), 158.58 (C=O of amide), 138.08 (CH of ethylene), 134.81 (C of phenyl), 122.26 (CH of phenyl), 130.82 (CH of phenyl), 115.22 (CH of phenyl), 158.49 (C of phenyl), 118.28 (CH of phenyl), 122.34 (CH of ethylene), 24.11 (CH_2_, diazepane), 40.02 (CH_2_, diazepane), 32.63 (CH_2_ of COCH_2_CH_3_), 24.04 (CH_3_ of COCH_2_CH_3_); MS ES + (ToF): m/z 362.3.

#### 4-Benzoyl-*N*-hydroxy-1-(3-(3-hydroxyphenyl)acryloyl)- 1,4-diazepane-2-carboxamide (7)

Mp (°C) 230–231; Yield—66.5%; IR (KBr pellets, cm^−1^): 1172 (C–O), 1288 (C–N), 1396 (C–H), 1423 (CH3), 1581 (C=C), 1676 (C=N), 1793 (C=O), 2090 (C≡C), 2241 (C≡N), 2843 (C–H), 2910 (=C–H), 3030 (=C–H), 3155 (≡C–H), 3423 (N–H), 3770 (O–H); ^1^H NMR (DMSO-*d*_6_, δ ppm): 2.016 (s, 1H, OH of NHOH), 8.121 (s, 1H, NH of CONHOH), 5.361 (H, OH, aromatic), 1.891–5.012 (m, 9H, diazepane), 6.945–7.535 (m, 9H, CH, aromatic), 7.334 (s, 1H, CH of ethylene), 6.985 (s, 1H, CH of ethylene); ^13^CNMR (DMSO-*d*_6_, δ ppm): 167.42 (C=O of COC_6_H_5_), 167.83 (C=O of CONHOH), 166.36 (C=O of amide), 141.38 (CH of ethylene), 134.36 (C of phenyl), 124.09 (CH of phenyl), 148.22 (C of phenyl), 122.12 (CH of phenyl), 130.79 (CH of phenyl), 122.12 (CH of phenyl), 134.81 (C of COC_6_H_5_), 124.15 (CH of COC_6_H_5_), 130.82 (CH of COC_6_H_5_), 130.82 (CH of COC_6_H_5_), 122.53 (CH of COC_6_H_5_), 122.34 (CH of COC_6_H_5_), 130.79 (CH of ethylene), 40.08 (CH, diazepane), 39.52 (CH_2_, diazepane), 39.02 (CH_2_, diazepane), 40.01 (CH_2_, diazepane); MS ES + (ToF): m/z 410.4.

#### 4-Butyryl-*N*-hydroxy-1-(3-(3-hydroxyphenyl)acryloyl)-1,4-diazepane-2-carboxamide (8)

Mp (°C) 255–255.5; Yield—67.3%; IR (KBr pellets, cm^−1^): 1170 (C–O), 1278 (C–N), 1400 (C–H), 1581 (C=C), 1622 (C=N), 1737 (C=O), 2086 (C≡C), 2241 (C≡N), 2935 (C–H), 2978 (=C–H), 3047 (=C–H), 3182 (≡C–H), 3427 (N–H), 3770 (O–H); ^1^H NMR (DMSO-*d*_6_, δ ppm): 2.002 (s, 1H, OH of NHOH), 8.112 (s, 1H, NH of CONHOH), 4.984 (H, OH, aromatic), 2.732–5.161 (m, 9H, diazepane), 7.235–7.523 (m, 4H, CH of C_6_H_5_), 7.334 (s, 1H, CH of ethylene), 7.176 (s, 1H, CH of ethylene), 2.712 (m, 2H, CH_2_ of COC_3_H_7_), 1.011 (m, 3H, CH_3_ of COC_3_H_7_); ^13^CNMR (DMSO-*d*_6_, δ ppm): 167.72 (C=O of COC_3_H_7_), 168.25 (C=O of CONHOH), 158.58 (C=O of amide), 138.08 (CH of ethylene), 134.81 (C of phenyl), 116.71 (CH of phenyl), 158.49 (C of phenyl), 115.22 (CH of phenyl), 130.29 (C of phenyl), 118.28 (CH of phenyl), 130.79 (CH of ethylene), 40.06 (CH of diazepane), 24.04 (CH_2_ of diazepane), 32.63 (CH_2_ of diazepane), 32.70 (CH_2_ of COC_3_H_7_), 24.11 (CH_2_ of COC_3_H_7_), 18.28 (CH_3_ of COC_3_H_7_).

#### 4-Acetyl-*N*-hydroxy-1-(3-(4-hydroxyphenyl)acryloyl)-1,4-diazepane-2-carboxamide (9)

Mp (°C) 124–125; Yield—68.1%; IR (KBr pellets, cm^−1^): 1172 (C–O), 1276 (–C–N), 1433 (–C–H), 1581 (C=C), 1627 (C=N), 1732 (C=O), 2135 (C≡C), 2239 (C≡N), 2677 (–C–H=O), 2742 (–C–H = O), 2935 (C–H), 2970 (=C–H), 3059 (=C–H), 3167 (≡C–H), 3414 (N–H), 3504 (O–H), 3753 (O–H); ^1^H NMR (DMSO-*d*_6_, δ ppm): 2.010 (s, 1H, OH of NHOH), 8.110 (s, 1H, NH of CONHOH), 5.462 (s, H, aromatic OH), 2.761–5.161 (m, 9H, diazepane), 6.562–7.534 (m, 4H, CH of C_6_H_5_), 7.334 (s, 1H, CH of ethylene), 7.217 (s, 1H, CH of ethylene), 2.712 (m, 3H, CH_3_ of COCH_3_); ^13^CNMR (DMSO-*d*_6_, δ ppm): 168.25 (C=O of COCH_3_), 167.72 (C=O of CONHOH), 158.58 (C=O of amide), 138.08 (CH of ethylene), 124.15 (C of phenyl), 130.79 (CH of phenyl), 116.71 (CH of phenyl), 158.49 (C of phenyl), 115.22 (CH of phenyl), 130.82 (CH of phenyl), 122.53 (CH of ethylene), 24.11 (CH_2_, diazepane), 40.02 (CH_2_, diazepane), 26.95 (CH_3_ of COCH_3_).

#### *N*-hydroxy-1-(3-(4-hydroxyphenyl)acryloyl)-4-propionyl-1,4-diazepane-2-carboxamide (10)

Mp (°C) 135; Yield—69.1%; IR (KBr pellets, cm^−1^): 1172 (C–O), 1400 (–C–H), 1581 (C=C), 1622 (C=N), 1732 (C=O), 2140 (C≡C), 2245 (C≡N), 2681 (–C–H=O), 2735 (–C–H=O), 2937 (C–H), 2953 (C–H), 2999 (=C–H), 3043 (=C–H), 3161 (≡C–H), 3400 (N–H), 3429 (N–H), 3522 (O–H), 3770 (O–H); ^1^H NMR (DMSO-*d*_6_, δ ppm): 2.010 (s, 1H, OH of NHOH), 8.110 (s, 1H, NH of CONHOH), 5.462 (s, H, OH, aromatic), 2.761–5.161 (m, 9H, diazepane), 6.562–7.534 (m, 4H, CH of C_6_H_5_), 7.334 (s, 1H, CH of ethylene), 7.217 (s, 1H, CH of ethylene), 2.712 (m, 2H, CH_2_ of COC_2_H_5_), 2.712 (m, 3H, CH_3_ of COC_2_H_5_); ^13^CNMR (DMSO-*d*_6_, δ ppm): 168.25 (C=O of COC_2_H_5_), 167.72 (C=O of CONHOH), 158.58 (C=O of amide), 138.08 (CH of ethylene), 124.15 (C of phenyl), 130.79 (CH of phenyl), 118.71 (CH of phenyl), 158.49 (C of phenyl), 115.22 (CH of phenyl), 130.79 (CH of phenyl), 122.53 (CH of ethylene), 40.04 (CH, diazepane), 39.42 (CH_2_, diazepane), 32.70 (CH_2_, diazepane), 24.04 (CH_2_, diazepane), 32.60 (CH_2_, diazepane), 24.11 (CH_2_ of COC_2_H_5_).

#### 4-Benzoyl-*N*-hydroxy-1-(3-(4-hydroxyphenyl)acryloyl)-1,4-diazepane-2-carboxamide (11)

Mp (°C) 232–233; Yield—68.1%; IR (KBr pellets, cm^−1^):1174 (C–O), 1288 (C–N), 1425 (C–H), 1581 (C=C), 1616 (C=N), 1629 (C=N), 1699 (C=O), 1791 (C=O), 1928 (–C–H), 2129 (C≡C), 2241 (C≡N), 2735 (–C=O–OH), 2958 (C–H), 2987 (C–H), 3018 (=C–H), 3062 (=C–H), 3176.76 (≡C–H), 3456 (N–H), 3469 (N–H), 3755 (O–H), 3770 (O–H); ^1^H NMR (DMSO-*d*_6_, δ ppm): 2.013 (s, 1H, OH of NHOH), 8.104 (s, 1H, NH of CONHOH), 4.985 (s, H, OH, aromatic), 1.705–5.215 (m, 9H, diazepane), 6.945–7.643 (m, 9H, CH, aromatic), 7.334 (s, 1H, CH of ethylene), 7.176 (s, 1H, CH of ethylene); ^13^CNMR (DMSO-*d*_6_, δ ppm): 168.25 (C=O of COC_6_H_5_), 167.72 (C=O of CONHOH), 158.58 (C=O of amide), 138.08 (CH of ethylene), 124.15 (C of phenyl), 130.79 (CH of phenyl), 116.71 (CH of phenyl), 158.49 (C of phenyl), 115.22 (CH of phenyl), 130.79 (CH of phenyl), 134.81 (C of COC_6_H_5_), 124.34 (CH of COC_6_H_5_), 130.82 (CH of COC_6_H_5_), 132.34 (CH of COC_6_H_5_), 130.82 (CH of COC_6_H_5_), 122.34 (CH of COC_6_H_5_), 124.15 (CH of ethylene), 40.04 (CH, diazepane), 39.82 (CH_2_, diazepane).

#### 4-Butyryl-*N*-hydroxy-1-(3-(4-hydroxyphenyl)acryloyl)-1,4-diazepane-2-carboxamide (12)

Mp (°C) 251–252; Yield—67.8%; IR (KBr pellets, cm^−1^): 1170 (C–O), 1325 (C–N), 1431 (C–H), 1581 (C=C), 1622 (C=N), 1722 (C=O), 1732 (C=O), 2102 (C=O), 2135 (C≡C), 2243 (C≡N), 2742 (–C=O–OH), 2939 (C–H), 2974 (=C–H), 3390 (≡C–H), 3419 (N–H), 3444 (N–H), 3743 (O–H); ^1^H NMR (DMSO-*d*_6_, δ ppm): 2.010 (s, 1H, OH of NHOH), 8.110 (s, 1H, NH of CONHOH), 4.910 (s, H, OH, aromatic), 1.171–5.161 (m, 9H, diazepane), 6.562–7.523 (m, 4H, CH, aromatic), 7.334 (s, 1H, CH of ethylene), 6.981 (s, 1H, CH of ethylene), 2.712 (m, 2H, CH_2_ of COC_3_H_7_), 1.167 (m, 3H, CH_3_ of COC_3_H_7_); ^13^CNMR (DMSO-*d*_6_, δ ppm): 168.25 (C=O of COC_3_H_7_), 167.72 (C=O of CONHOH), 158.58 (C=O of amide), 138.08 (CH of ethylene), 124.15 (C of phenyl), 130.79 (CH of phenyl), 118.71 (CH of phenyl), 158.49 (C of phenyl), 115.22 (CH of phenyl), 130.79 (CH of phenyl), 122.53 (CH of ethylene), 40.04 (CH, diazepane), 32.70 (CH_2_, diazepane), 24.04 (CH_2_, diazepane), 32.63 (CH_2_ of COC_3_H_7_), 24.11 (CH_2_ of COC_3_H_7_), 18.28 (CH_3_ of COC_3_H_7_).

#### 4-Acetyl-*N*-hydroxy-1-(3-(3,4-dihydroxyphenyl)acryloyl)-1,4-diazepane-2-carboxamide (13)

Mp (°C) 180; Yield—68.1%; IR (KBr pellets, cm^−1^):1172 (C–O), 1263 (C–N), 1440 (C–H), 1581 (C=C), 1622 (C=N), 1793 (C=O), 2129 (C≡C), 2241 (C≡N), 2677 (–C=O–OH), 2935 (C–H), 2976 (C–H), 3057 (=C–H), 3101 (=C–H), 3149 (≡C–H), 3161 (≡C–H), 3462 (N–H), 3481 (N–H), 3755 (O–H); ^1^H NMR (DMSO-*d*_6_, δ ppm): 2.010 (s, 1H, OH of NHOH), 8.091 (s, 1H, NH of CONHOH), 4.918 (d, 2H, aromatic OH), 2.873–5.161 (m, 9H, diazepane), 6.945–7.951 (m, 3H, CH of C_6_H_5_), 7.446 (s, 1H, CH of ethylene), 6.985 (s, 1H, CH of ethylene), 2.780 (m, 3H, CH_3_ of COCH_3_); ^13^CNMR (DMSO-*d*_6_, δ ppm): 168.25 (C=O of COCH_3_), 167.72 (C=O of CONHOH), 158.58 (C=O of amide), 138.08 (CH of ethylene), 130.79 (C of phenyl), 116.71 (CH of phenyl), 158.49 (C of phenyl), 158.58 (C of phenyl), 118.28 (CH of phenyl), 122.53 (CH of phenyl), 124.15 (CH of ethylene), 24.11 (CH_2_, diazepane), 40.08 (CH, diazepane), 26.95 (CH_3_ of COCH_3_).

#### *N*–hydroxy-1-(3-(3,4-dihydroxyphenyl)acryloyl)-4-propionyl-1,4-diazepane-2-carboxamide (14)

Mp (°C) 200; Yield—69.2%; IR (KBr pellets, cm^−1^): 1170 (C–O), 1263 (C–N), 1431 (C–H), 1581 (C=C), 1645 (C=N), 1720 (C=O), 2113 (C≡C), 2306 (C≡N), 2692 (–C=O–OH), 2893 (C–H), 3022 (=C–H), 3167 (≡C–H), 3265 (≡C–H), 3433 (N–H), 3471 (N–H), 3520 (O–H), 3709 (O–H); ^1^H NMR (DMSO-*d*_6_, δ ppm): 2.010 (s, 1H, OH of NHOH), 8.110 (s, 1H, NH of CONHOH), 4.910 (d, 2H, aromatic OH), 2.765–5.161 (m, 9H, diazepane), 6.562–7.523 (m, 3H, CH of C_6_H_5_), 7.334 (s, 1H, CH of ethylene), 6.981 (s, 1H, CH of ethylene), 2.712 (m, 2H, CH_2_ of COC_2_H_5_), 1.171 (m, 3H, CH_3_ of COC_2_H_5_); ^13^CNMR (DMSO-*d*_6_, δ ppm): 168.25 (C=O of COC_2_H_5_), 167.72 (C=O of CONHOH), 158.58 (C=O of amide), 138.08 (CH of ethylene), 130.82 (C of phenyl), 116.71 (CH of phenyl), 158.49 (C of phenyl), 158.58 (C of phenyl), 118.28 (CH of phenyl), 124.15 (CH of phenyl), 122.53 (CH of ethylene), 40.04 (CH, diazepane), 32.63 (CH_2_, diazepane), 24.04 (CH_2_, diazepane), 24.11 (CH_2_ of COC_2_H_5_); MS ES + (ToF): m/z 378.4.

#### 4-Benzoyl-*N*-hydroxy-1-(3-(3,4-dihydroxyphenyl)acryloyl)-1,4-diazepane-2-carboxamide (15)

Mp (°C) 240–242; Yield—69.3%; IR (KBr pellets, cm^−1^): 1168 (C–O), 1205 (C–N), 1263 (C–H), 1323 (C=C), 1394 (C=N), 1440 (C=O), 1469 (C≡C), 1581 (C≡N), 1637 (–C=O–OH), 1728 (C–H), 1843 (=C–H), 1865 (≡C–H), 2133 (≡C–H), 2243 (N–H), 2306 (N–H), 2353 (O–H), 2490 (O–H); ^1^H NMR (DMSO-*d*_6_, δ ppm): 2.197 (s, 1H, OH of NHOH), 8.123 (s, 1H, NH of CONHOH), 4.731 (d, 2H, aromatic OH), 2.780–5.012 (m, 9H, diazepane), 6.945–7.535 (m, 8H, CH, aromatic), 7.334 (s, 1H, CH of ethylene), 6.985 (s, 1H, CH of ethylene); ^13^CNMR (DMSO-*d*_6_, δ ppm): 168.25 (C=O of COC_6_H_5_), 167.72 (C=O of CONHOH), 158.58 (C=O of amide), 138.08 (CH of ethylene), 130.82 (C of phenyl), 116.71 (CH of phenyl), 158.49 (CH of phenyl), 158.58 (C of phenyl), 118.28 (CH of phenyl), 122.53 (CH of phenyl), 124.15 (CH of ethylene), 40.04 (CH, diazepane); MS ES + (ToF): m/z 427.5.

#### 4-Butyryl-*N*-hydroxy-1-(3-(3,4-dihydroxyphenyl)acryloyl)-1,4-diazepane-2-carboxamide (16)

Mp (°C) 266; Yield—68.9%; IR (KBr pellets, cm^−1^): 1163 (C–O), 1296 (–C–N), 1440 (–C–H), 1581 (C=C), 1637 (C=N), 1705 (C=O), 1716 (C=O), 2133 (C≡C), 2245 (C≡N), 2675 (–C–H=O), 2744 (–C–H=O), 2935 (C–H), 2970 (=C–H), 3064 (=C–H), 3246 (≡C–H), 3408 (N–H), 3755 (O–H); ^1^H NMR (DMSO-*d*_6_, δ ppm): 2.010 (s, 1H, OH of NHOH), 8.110 (s, 1H, NH of CONHOH), 4.910 (d, 2H, aromatic OH), 1.171–5.161 (m, 9H, diazepane), 6.981–7.523 (m, 3H, CH, aromatic), 7.334 (s, 1H, CH of ethylene), 7.217 (s, 1H, CH of ethylene); ^13^CNMR (DMSO-*d*_6_, δ ppm): 168.25 (C=O of COC_3_H_7_), 167.72 (C=O of CONHOH), 158.58 (C=O of amide), 138.08 (CH of ethylene), 130.82 (C of phenyl), 116.71 (CH of phenyl), 158.49 (CH of phenyl), 158.58 (C of phenyl), 118.28 (CH of phenyl), 122.53 (CH of phenyl), 122.53 (CH of ethylene), 40.04 (CH, diazepane), 24.04 (CH_2_, diazepane), 24.11 (CH_2_ of COC_3_H_7_).

### Biological evaluation

#### Cell culture

Dulbecco’s Modified Eagle Medium (DMEM), Penicillin and Streptomycin purchased from Himedia, Mumbai; Fetal Calf Serum (FCS) from Lonza, Belgium; DMSO (dimethyl sulfoxide) from Sigma-Aldrich, USA; MTT reagent [3-(4,5-dimethylthiazol-2-yl)-2, 5-diphenyltetrazolium bromide] from Merck, India and Paclitaxel from Dabur, India. Cell lines were procured from National Centre for Cell Science (NCCS), Pune, India. A549 human lung cancer cells were grown in DMEM supplemented with 100 U/mL, Penicillin G, 100 lg/mL Streptomycin, 0.25 lg/mL, Amphotericin, and 10% heat inactivated fetal bovine serum. Cultures were maintained at 37 °C in a 5% CO2, 95% air atmosphere.

#### In vitro anticancer assays

##### MTT assay

Preliminary cytotoxic activity was assayed by MTT assay as previously described [26]. In brief, A549 cell lines were grown for 48 h after incubation at various concentrations of synthesized compounds. The optical density (OD) was measured by ELISA plate reader at 570 nm with a reference wavelength of 630 nm. OD was expressed as percentage cell survival (absorbance of treated wells/absorbance of control wells × 100). Results were expressed as Mean ± S.E. and based on the results; the active compounds were considered to be significant which gave less than 50% survival at the exposure time of 48 h.

##### Semi-quantitative RT-PCR (mRNA analysis)

Based on preliminary cytotoxicity results, gene expression was assayed by semi-quantitative RT-PCR as previously described [37]. In short, cancer cells (2 × 10^6^ cells/mL) were treated with selected active compounds for 18 h followed by isolation of total RNA and then quantification. After that, 1 µg of total RNA was used for cDNA synthesis. The cDNA amplification was done with gene specific primers: “human MMP-2 (5′-GTG CTG AAG GAC ACA CTA AAG AAG A-3′, 3′-GGA TGT TGA AAC TCT TCC TAC CGT T-5′); MMP-9 (5′-CAC TGT CCA CCC CTC AGA GC-3′, 3′-GGA ATA GCG GCT GTT CAC CG-5′), β-actin (5′-TGT GAT GGT GGG AAT GGG TCA G-3′, 5′-TTT GAT GTC ACG CAC GAT TTC C-3′). β-Actin primers were used as normalization control. The PCR products were separated on a 2% agarose gel containing ethidium bromide (0.5 µg/mL), visualized, and photographed using a gel documentation system.

### Molecular docking studies

The docking studies were performed for selected compounds (6, **7**, **8**, **15** and **16**) in the binding site of MMP-2 and MMP-9 proteins (PDB entries: 1HOV and 4H3X respectively) using AutoDock Vina [23, 32] and graphical user interface, Auto-dock tools installed on windows 7 [17]. The X-ray crystallographic information of MMP-2 and MMP-9 proteins was acquired from protein data bank (http://www.rcsb.org/pdb) and after evaluation of a number of entries, the best X-ray structures were chosen by analyzing the proteins with the highest resolution. The PDB file of MMP-2 and MMP-9 proteins was edited with the help of PyMOL, and α chain was removed along with the complexed inhibitor. All interacting ions and water molecules were removed. The PDBQT file for the proteins was generated with the help of AutoDock tools by addition of all polar hydrogen atoms charge assignment to the macromolecule. The geometries of the ligands were optimized by Open Babel using force field [20]. The ligands were prepared for docking by using AutoDock tools by assigning the charges to all the atoms and storing them as PDBQT file. The calculations of grid parameters were accomplished by using the Grid tool in Auto-Dock tools. The grid parameter file possessing all information regarding the protein, size of grid, geometry of search space and ligand was built and was kept as ‘Conf.txt’.

The docking of co-crystallized inhibitors into the active site of target proteins was executed for the determination of accuracy of docking protocol. The optimized ligand molecules in PDBQT format were docked in the active site of MMP-2 and MMP-9 proteins by means of AutoDock Vina. Docking runs were launched from the command line, followed by the generation and scoring of best nine poses, for each and every ligand using AutoDock Vina scoring function. At the end of the docking, ligands with utmost favorable free energy (− ΔG) of binding were carefully chosen. “Lower is the value; higher is the interaction, thus, stability of the ligand–protein complex”. The hydrophobic, hydrogen bond and other interactions were further analyzed for the docked ligands by using PyMOL and best poses in the binding site were drawn.

## Results

### Chemistry

**Fig. 2:**
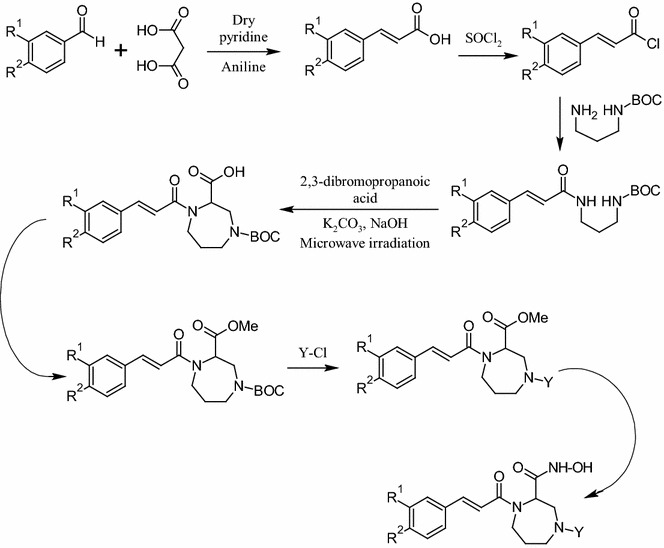
Synthesis of diazepine substituted cinnamic acid derivatives

The substituted cinnamic acid derivatives were synthesized by the synthetic route as highlighted in Fig. 2. In the first step, substituted benzaldehyde derivatives and malonic acid were reacted to form cinnamic acid derivatives. In the second step, the corresponding cinnamic acid derivatives were reacted with tert-butyl (3-aminopropyl)carbamate to synthesize tert-butyl (3-cinnamamidopropyl)carbamate derivatives. The tert-butyl (3-cinnamamidopropyl)carbamate derivatives were reacted with 2,3-dibromopropanoic acid and potassium carbonate using a microwave synthesizer (Temp. 120 °C, 90 W Power, and 20 min reaction time) resulting in 4-(tert-butoxycarbonyl)-1-cinnamoyl-1,4-diazepane-2-carboxylic acid derivatives. This step was followed by reaction with acyl and aryl chlorides to obtain diazepine substituted cinnamic acid derivatives. In the last step the diazepine substituted cinnamic acid derivatives were reacted with hydroxylamine to get the desired molecules. The physicochemical characteristics of the synthesized compounds are presented in Table 1. The synthesized compounds were characterized by FTIR, ^1^H and ^13^C NMR, and Mass spectra and the results were in accord with the allocated molecular structures.

**Table 1:** Physiochemical properties of synthesized diazepine substituted cinnamic acid derivatives and cytotoxicity evaluation on A549 cell lines

C. no.	R1, R2	Y	Mol. wt	Rf value^a^	M. Pt.	IC_50_ (µM)^b^
1	H, H	–COC_6_H_5_	393.443	0.57	210	13.1 ± 0.2
2	H, H	–COCH_2_CH_2_CH_3_	359.426	0.51	121–123	12.5 ± 0.9
3	H, H	–COCH_3_	331.372	0.46	164	17.8 ± 0.5
4	H, H	–COCH_2_CH_3_	345.399	0.55	190	13.5 ± 0.7
5	3-OH	–COCH_3_	347.371	0.47	123	13.9 ± 0.6
6	3-OH	–COCH_2_CH_3_	361.398	0.52	125–127	11.2 ± 0.6
7	3-OH	–COC_6_H_5_	409.442	0.50	230–231.5	8.5 ± 0.8
8	3-OH	–COCH_2_CH_2_CH_3_	375.425	0.60	255–255.3	7.7 ± 0.5
9	4-OH	–COCH_3_	347.371	0.56	124–125	13.6 ± 0.4
10	4-OH	–COCH_2_CH_3_	361.398	0.80	135	12.8 ± 0.9
11	4-OH	–COC_6_H_5_	409.442	0.48	232–233	11.1 ± 0.7
12	4-OH	–COCH_2_CH_2_CH_3_	375.425	0.30	251–252	10.3 ± 0.7
13	3, 4-di OH	–COCH_3_	363.370	0.75	180	11.8 ± 0.6
14	3, 4-di OH	–COCH_2_CH_3_	377.397	0.40	200	10.1 ± 0.5
15	3, 4-di OH	–COC_6_H_5_	425.441	0.65	240–242	8.2 ± 0.7
16	3, 4-di OH	–COCH_2_CH_2_CH_3_	391.424	0.75	266	7.3 ± 0.3
Pac^c^	–	–	–	–	–	7.3 ± 0.7

^a^Mobile phase: dichloromethane: methanol (19:1)

^b^Mean ± S.D. (n = 3)

^c^Pac—Paclitaxel

### MTT assay

The anticancer potential of the synthesized compounds was measured (**1**–**16**) by MTT assay. The results indicated that all the synthesized compounds were found to be active against A549 cancer cells and showed dose-dependent cytotoxicity. Data also pointed out that amongst the synthesized compounds; compounds **8** (IC50 = 7.7 ± 0.5 µM) and **16** (IC50 = 7.3 ± 0.3 µM) showed comparable cytotoxicity in comparison with standard (paclitaxel—IC50 = 7.3 ± 0.7 µM) [2, 6, 9, 15, 21, 35, 38]. Compounds **7** (IC50 = 8.5 ± 0.8 µM), and **15** (8.2 ± 0.7 µM) also showed considerable activity against the cancer cell lines (Table 1). Compound **16** was found to be most potent against A549 cells.

### Selected compounds downregulates the expressions of MMPs (-2 and -9)

**Fig. 3:**
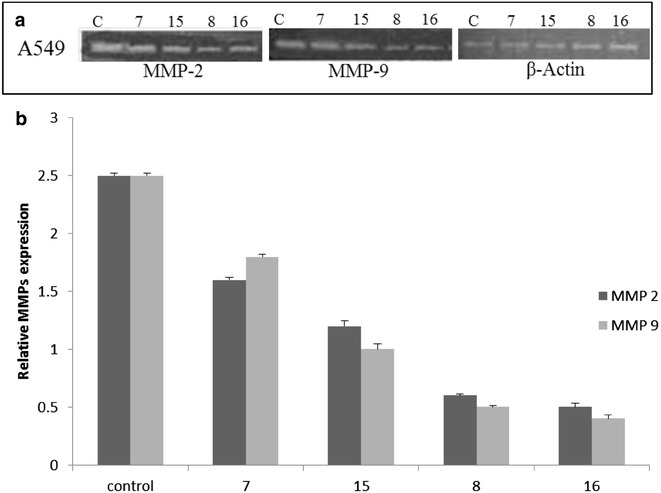
**a**, **b** Relative mRNA expression of gelatinases MMPs shows a down regulation in collagen degrading enzymes upon treatment. **c** Control; Compounds 7 (8.5 µM), 8 (7.7 µM), 15 (8.2 µM), and 16 (7.3 µM). Data are illustrative of a minimum of three independent experiments

MMPs (-2 and -9) have been indicated to be associated with cancer metastasis, we, therefore, investigated whether the compounds 7, 8, 15 and 16 were involved in invasion down regulation. It was confirmed by the inhibition of MMPs activity by RT-PCR (m-RNA analysis) method by measuring the expression levels of MMP-2 and MMP-9. We found that compounds 8 and 16 significantly inhibited MMP-2 and MMP-9 activity in A549 cells; however the inhibition of MMP-2 and MMP-9 activity by compounds 7 and 15 was comparatively less. Compounds 8 and 16 significantly suppressed the expression of MMP-2 and MMP-9 protein and mRNA levels (Fig. 3a, b) which forms a specific complex with the MMPs and thus inhibits the activation of MMP-2 and MMP-9. The results indicated that compounds 8 and 16 have the tendency to inhibit the metastasis of cancer. Based on the results, it can be concluded that compound **16** may be taken as a lead compound for the discovery of new drug molecules for the treatment of lung cancer.

### Molecular docking

Lead optimization of the synthesized compounds was done by computation of drug-likeness properties molecular weight, partition coefficient i.e., log P, hydrogen bond donors (HBA), and hydrogen bond acceptors (HBD). Most of the selected compounds for in silico studies were found to possess drug like properties as derived by Lipinski’s rule of five. Docking studies were carried out to evaluate the affinity and binding interactions of the selected synthesized molecules in the active site of MMP-2 (PDB entry: 1HOV) and MMP-9 (PDB entry: 4H3X) proteins using AutoDock Vina and the graphical user interface, AutoDockTools installed on Windows 7. The docking protocol was validated by docking of co-crystallized ligand into the active site, and the resulting binding pose was compared with that of reference ligands (MMP-2: *N*-{4-[(1-hydroxycarbamoyl-2-methyl-propyl)-(2-morpholin-4-yl-ethyl)-sulfamoyl]-4-pentyl-benzamide; MMP-9: *N*-2-(biphenyl-4-ylsulfonyl)-*N*-2-(isopropyloxy)-acetohydroxamic acid). The ligands had a similar binding pattern and superposition to that of co-crystallized ligands, thus validating the docking protocol. The selected compounds showed appreciable binding to the binding site of MMP-2 and MMP-9 proteins as determined by analyzing their bonding interactions in terms of H-bond, hydrophobic interactions and binding free energy (− ΔG, kcal/mol) of the selected best docked poses (Table 2). These compounds were further analyzed in details by Molecular Visualization Tool, PyMOL.

**Table 2:** Docking scores and molecular properties of selected compounds

Compound	Mol. wt^a^	HBA^a^	HBD^a^	Log P^a^	ΔG (MMP-2)*	ΔG (MMP-9)*
**7**	409.44	5	3	1.29	− 7.8	− 8.0
**8**	375.42	5	3	0.59	− 7.0	− 7.6
**15**	425.44	6	4	1.34	− 8.1	− 8.9
**16**	391.42	6	4	0.75	− 7.0	− 7.7

*ΔG (KJ/mol) for reference ligand: − 8.3 and − 8.5 for MMP-2 and MMP-9, respectively

^a^Mol. wt, HBA, HBD, and log P were calculated by Marvin tools of Marvin Sketch

#### MMP-2 overlays

##### MMP-2 overlays

**Fig. 4:**
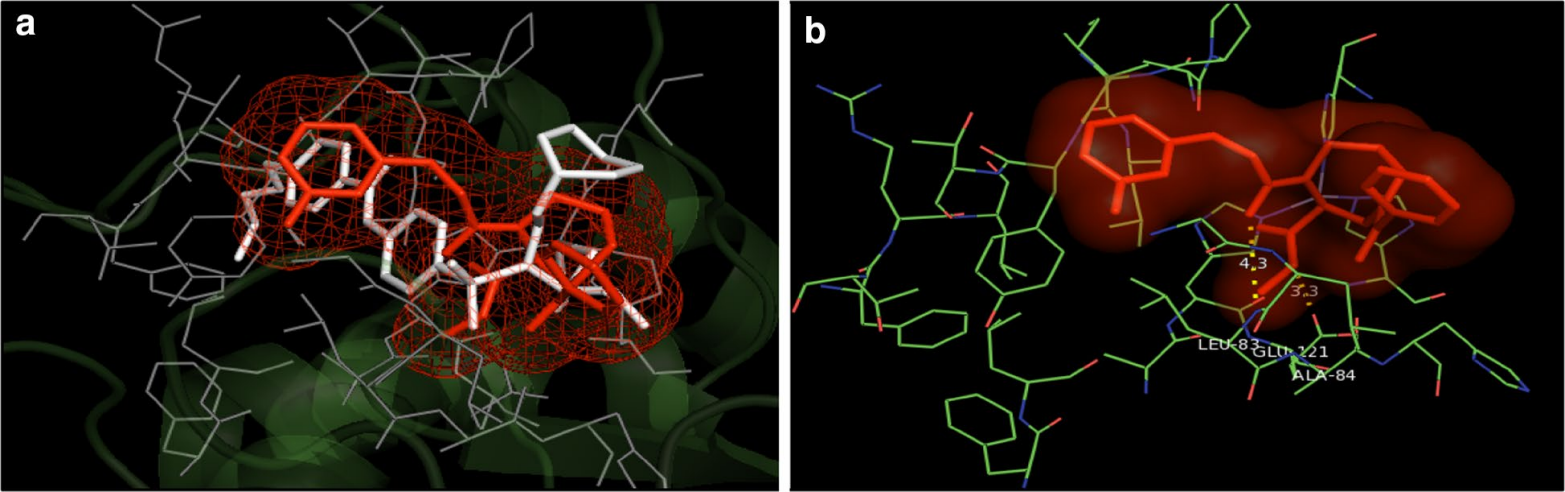
**a** Overlay of the docked pose of compound **7** (red) with that of PDB Ligand 1HOV (white); **b** docked pose: H-bond interaction of compound **7** in the binding site of MMP-2 protein

**Fig. 5:**
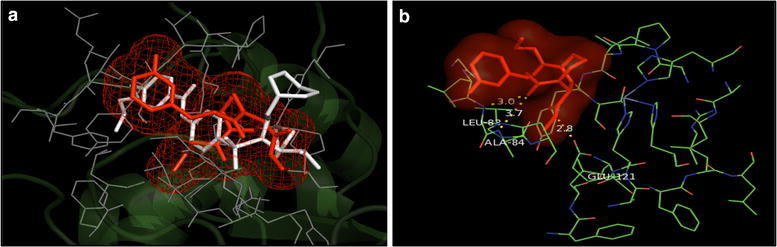
**a** Overlay of the docked pose of compound **8** (red) with that of PDB Ligand 1HOV (white); **b** docked pose: H-bond interaction of compound **8** in the binding site of MMP-2 protein

**Fig. 6:**
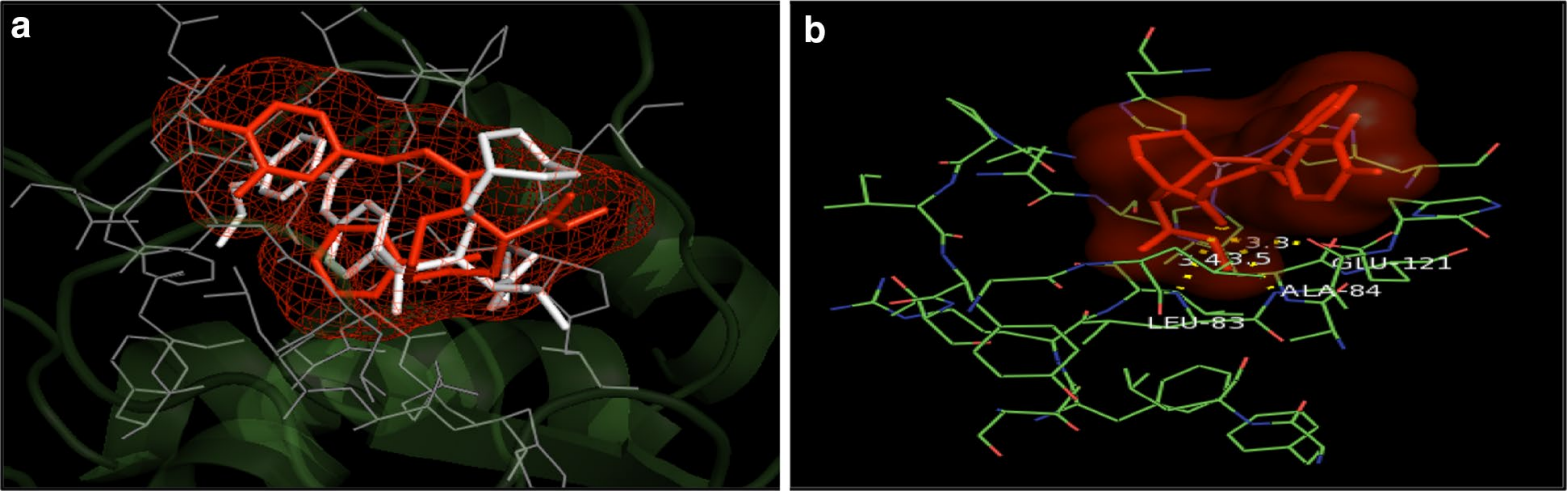
**a** Overlay of the docked pose of compound **15** (red) with that of PDB Ligand 1HOV (white); **b** docked pose: H-bond interaction of compound **15** in the binding site of MMP-2 protein

**Fig. 7:**
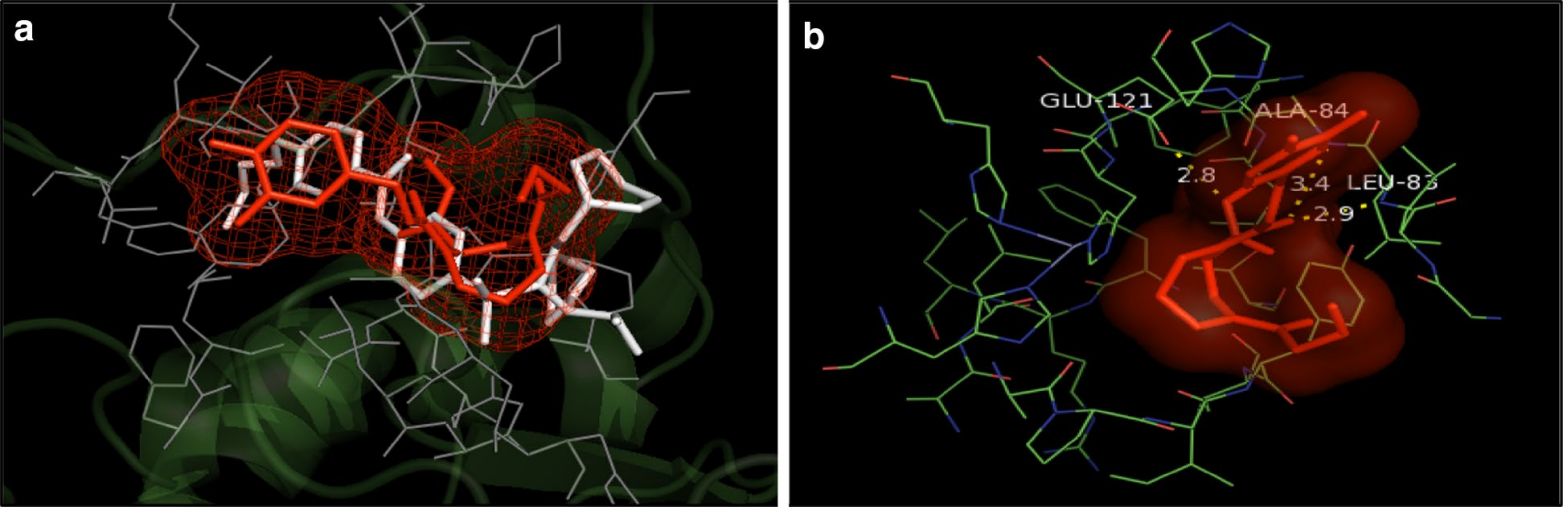
**a** Overlay of the docked pose of compound **16** (red) with that of PDB Ligand 1HOV (white); **b** docked pose: H-bond interaction of compound **16** in the binding site of MMP-2 protein

The overlay of docked poses of compounds **7**, **8**, **15** and **16** with that of 1HOV ligand showed that compounds **7**, **8**, **15** and **16** had similar binding pattern in the active site of MMP-2 protein as that of co-crystallized inhibitor (Figs. 4a, 5a, 6a and 7a). The docked pose of compound **7** showed the H-bond interaction between NH of NHOH and carbonyl group with COOH of Glu121 residue and NH of Leu83 residue in the active site of MMP-2 protein with H-bond distances of 3.3 and 4.3 Å, respectively (Fig. 4b). The docked pose of compound **8** showed the H-bond interaction between carbonyl with NH of Leu83 and Ala84 residues (3.0 and 3.7 Å); and between NH of NHOH and COOH of Glu121 residue (2.8 Å) (Fig. 5b). The docked pose of compound **15** showed the H-bond interaction between carbonyl with NH of Leu83 and Ala84 residues (3.4 and 3.5 Å); and between NH of NHOH and COOH of Glu121 residue (3.3 Å) (Fig. 6b). The docked pose of compound **16** showed the H-bond interaction between carbonyl with NH of Leu83 and Ala84 residues (2.9 and 3.4 Å); and between NH of NHOH and COOH of Glu121 residue (2.8 Å) (Fig. 7b). All the selected compounds showed appreciable metal interaction between OH of NHOH and Zn^2+^ ion in the binding site of MMP-2 protein.

##### MMP-9 overlays

**Fig. 8:**
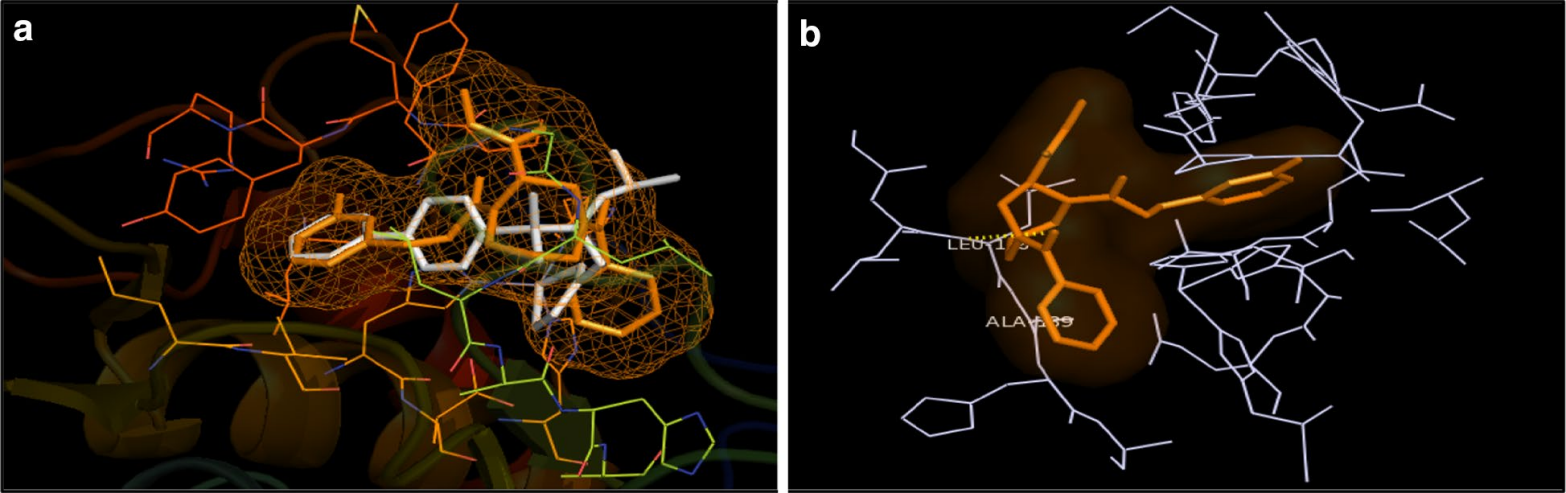
**a** Overlay of the docked pose of compound **7** (orange) with that of PDB Ligand 4H3X (white); **b** docked pose: H-bond interaction of compound **7** in the binding site of MMP-9 protein

**Fig. 9:**
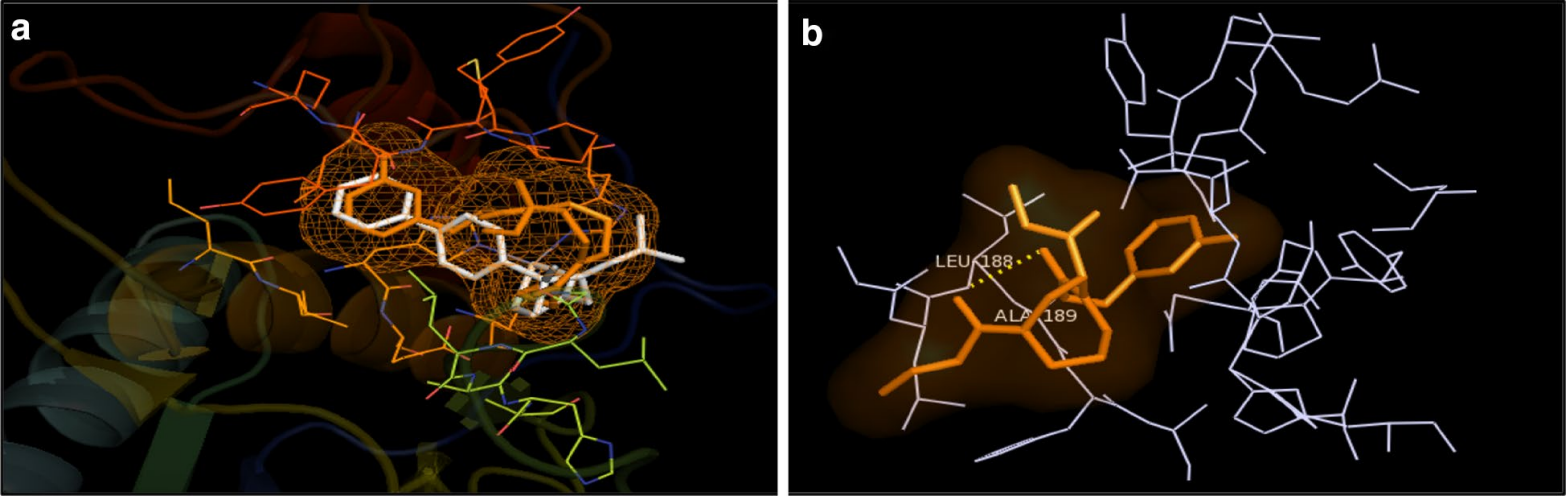
**a** Overlay of the docked pose of compound **8** (orange) with that of PDB Ligand 4H3X (white); **b** docked pose: H-bond interaction of compound **8** in the binding site of MMP-9 protein

**Fig. 10:**
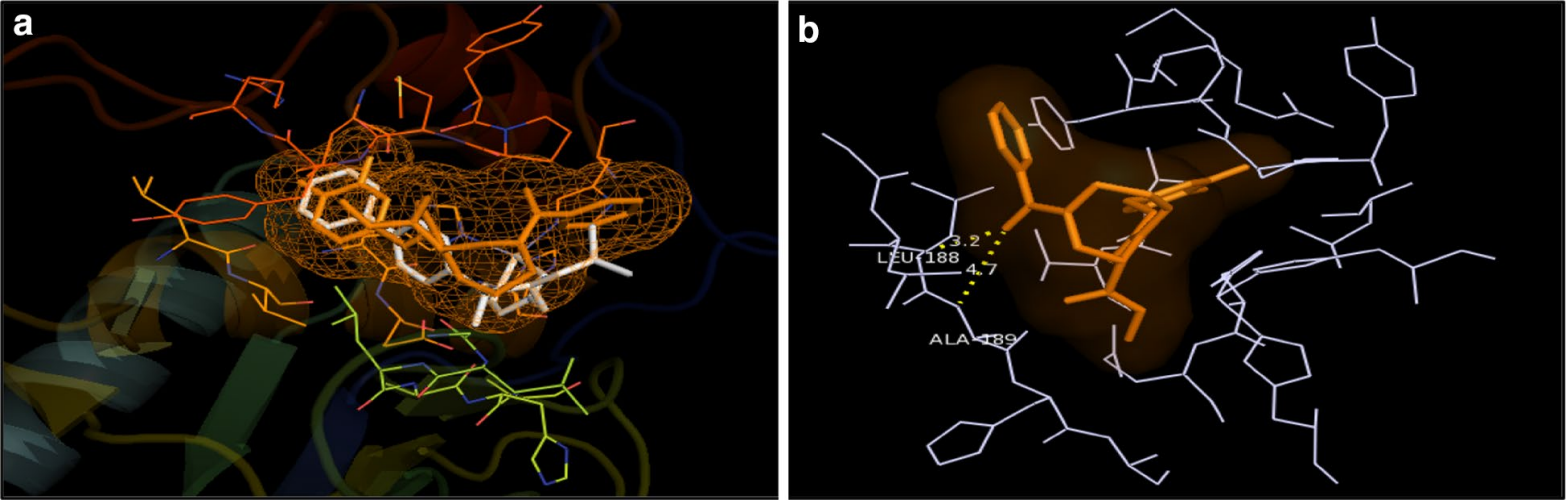
**a** Overlay of the docked pose of compound **15** (orange) with that of PDB Ligand 4H3X (white); **b** docked pose: H-bond interaction of compound **15** in the binding site of MMP-9 protein

**Fig. 11:**
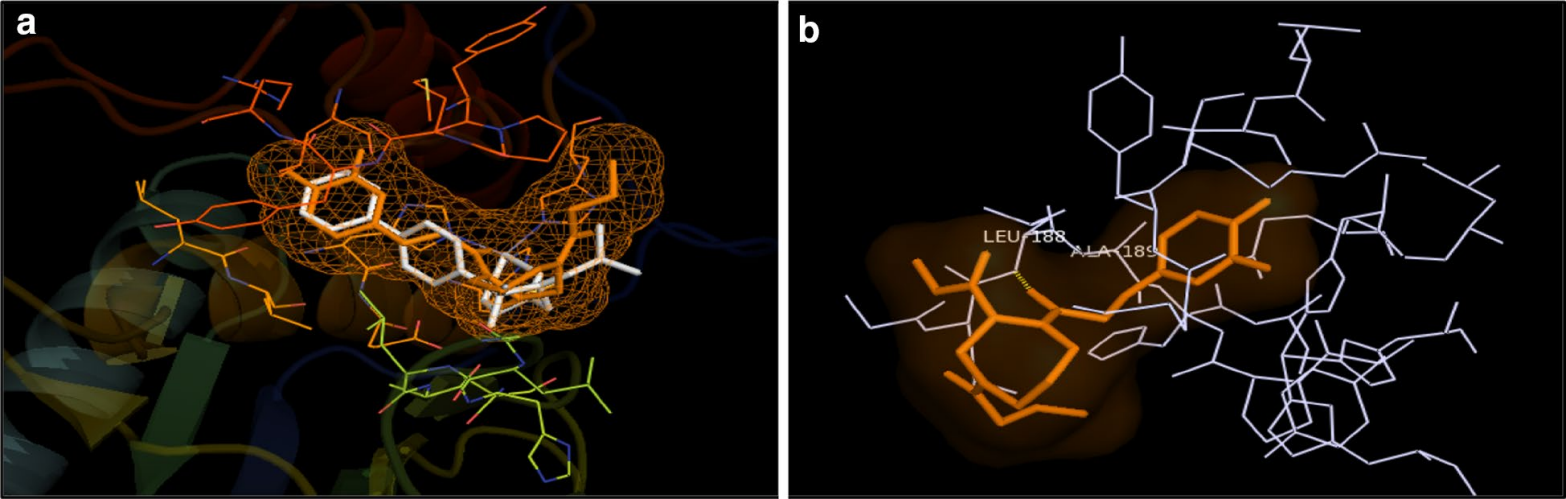
**a** Overlay of the docked pose of compound **16** (orange) with that of PDB Ligand 4H3X (white); **b** docked pose: H-bond interaction of compound **16** in the binding site of MMP-9 protein

The overlay of docked poses of compounds **7**, **8**, **15** and **16** with that of 4H3X ligand showed that the selected compounds had the similar binding pattern in the active site of MMP-9 protein as that of co-crystallized inhibitor (Figs. 8a, 9a, 10a and 11a). The docked pose of compound **7** showed a weak H-bond interaction between carbonyl and NH of Ala189 residue in the active site of MMP-9 protein (Fig. 8b). The docked pose of compound **8** showed a weak H-bond interaction between carbonyl and NH of Leu188 residue in the active site of MMP-9 protein (Fig. 9b). The docked pose of compound **15** showed appreciable H-bond interactions between carbonyl and NH of Leu188 and Ala189residues in the active site of MMP-9 protein (H-bond distance of 3.2 and 4.7 Å respectively) (Fig. 10b). The docked pose of compound **16** showed a weak H-bond between carbonyl group and NH of Leu188 residue (Fig. 11b). The hydroxamate group of all the docked compounds showed appreciable metal interaction with Zn^2+^ of the MMP-9 protein.

## Discussion

Metastasis is the leading reason for the resultant mortality of patients with cancer and is a major reason for treatment failure [13]. Previous reports have demonstrated that diazepine and caffeic acid (hydroxycinnamic acid) are correlated with MMPs inhibitory activity, however, there are no studies addressing anti-metastasis activity of diazepine substituted cinnamic acid derivatives. In the current study, a series of cinnamic acid derivatives clubbed with diazepine ring has been synthesized and characterized by physicochemical properties and spectral techniques. The synthesized compounds were screened for their in vitro anticancer potential and the results of cytotoxicity studies revealed that all the synthesized compounds were active against A549 cancer cell lines. Compounds **8** (IC50 7.7 µM) and **16** (IC50 = 7.3 µM) were found to be the most potent against the cancer lines. Further, it was proved that compounds 8 and 16 significantly suppressed the expression of MMP-2 and MMP-9 protein and mRNA levels which forms a specific complex with the MMPs and thus inhibits the activation of MMP-2 and MMP-9. The results indicated that compounds 8 and 16 have the tendency to inhibit the invasion and metastasis of cancer. Based on the results, it can be concluded that compound **16** may be taken as a lead compound for the discovery of new drug molecules for the treatment of lung cancer.

Based on the anticancer studies of synthesized diazepine substituted cinnamic acid derivatives, the following SAR can be concluded upon. Compounds 1–4 without hydroxyl groups (either 3 or 4 position) on the cinnamic acid showed least activity. This confirms the requirement of –OH groups at these positions. The presence of –OH groups at either 3 or 4 position of cinnamic acid increased the anticancer activity as see in case of compounds 5–16. Further the compounds having di-OH groups at both 3, 4 positions showed the most potent anticancer activity as observed in 16 and significantly inhibited the expression of MMPs and also decreased the invasive potential of A549 cells. These results showed that both the –OH groups were important for MMP inhibition and selectivity. The possible reason for this might be the solvent exposed region in the MMPs, which results in better binding. In addition, the presence of larger and extended groups like isobutyl group at N on 4-position of diazepine ring (i.e. the P1 site) significantly enhanced the anticancer activity and further the MMP inhibition and selectivity as in case of compounds 8 and 16. This is due to the fact that hydrophobic groups have strong hydrophobic interactions with MMP proteins.

In addition, the mode of interactions between compounds 7, 8, 15 and 16 with MMP-2 and MMP-9 were investigated through molecular docking studies and to find out a relationship between the anticancer properties of synthesized compounds and their structural features. Molecular docking study revealed that compounds 7, 8, 15 and 16 directly interacted with active site residues to inhibit MMP-2 and MMP-9 activities. There is evidence that MMP-2 has a channel-like S1’ cavity and MMP-9 has a pocket-like S1’ cavity with a floorboard [1, 12, 30] and the S1’ is a substrate binding pocket, formed by two subdomains. S1’ pocket is found in the center of active site cleft neighbouring to active site zinc ion and this pocket consists of Asp 185-Leu 188 and Pro 421-Tyr 423 which are accountable for hydrogen bonding to substrates/inhibitors. The hedge of S1’ cavity is formed by side chains of Leu 188, Leu 397, Val 398, His 401, Leu 418, and Met 422-Tyr 423 as main chain. Leu 397 and Val 398 are specific to MMP-9 [30]. Compounds **7**, **8**, **15** and **16** overlays showed similar binding pattern in the active site of MMP-2 protein as that of co-crystallized inhibitor. Further, docked poses of compounds 7, 8, 15 and 16, showed H-bond interaction: between NH of NHOH and carbonyl group with COOH of Glu 121 residue and NH of Leu 83residue (compound **7**); between carbonyl with NH of Leu 83 and Ala 84 residues and between NH of NHOH and COOH of Glu 121 (Compound **8**); between carbonyl with NH of Leu 83 and Ala 84and between NH of NHOH and COOH of Glu 121residue (compound **15**); between carbonyl with NH of Leu 83 and Ala 84 and between NH of NHOH and COOH of Glu 121 residues (compound **16**) in the active site of MMP-2 protein, respectively. All the selected compounds showed appreciable metal interaction between OH of NHOH and Zn^2+^ ion in the binding site of MMP-2 protein.

Further, the docked pose of compounds **7**, **8**, **15** and **16** showed H-bond interaction between H-bond interaction between carbonyl and NH of Ala189 residue (compound **7**), carbonyl and NH of Leu 188 residue (compound **8**); between carbonyl and NH of Leu 188 and Ala 189 residue (compound **15**); between carbonyl group and NH of Leu 188 residues (compound **16**) in the active site of MMP-9 protein, respectively. The hydroxamate group of all the docked compounds showed appreciable metal interaction with Zn^2+^ of the MMP-9 protein. In conclusion, the data strongly indicated that the selected compound **16** inhibit tumor invasion and migration by repressing MMP-2 and MMP-9 at protein and mRNA levels. Further, by directly interacting with active site residues to inhibit MMP-2 and MMP-9 activities. This study provides vital evidence about the anti-invasive potential of these prospecting candidates for developing into potential anticancer therapeutics, however further evidence should be conducted in in vivo studies to verify its application for clinical use in anti-metastatic effects on lung cancer cells.
